# Comment: Early nutrition prescription in critically ill patients—learnings from the FRANS study

**DOI:** 10.1186/s13054-023-04346-4

**Published:** 2023-02-20

**Authors:** Mette M. Berger, Pierre Singer

**Affiliations:** 1 Lausanne University Hospital (CHUV), University of Lausanne, 1011 Lausanne, Switzerland; 2 Department of Intensive Care, Institute for Nutrition Research, Rabin Medical Center, Beillison Hospital, Affiliated to the Sackler School of Medicine, Tel Aviv University, Tel Aviv, Israel

Once more a large clinical study confirms the importance of respecting physiology in the critically ill patient, observing the individual response, and administering feeding progressively whatever the route. The prospective observational ‘French-Speaking ICU Nutritional Survey’ (FRANS) study was conducted in 26 ICUs over 3 months in 2015 [1]: it investigated the impact on the 28-day outcome of the feeding strategy during the first 10 days of the intensive care (ICU) in 1206 patients. The authors should be commended for conducting this large study which provides further arguments in favour of a more physiological approach: it confirms that early high-energy feeding is deleterious in critically ill patients. Their study population is representative of critical illness (median SAPS II 44, SOFA score 8) with 81.2% intubated patients. Early nutrition support was administered to 718 patients (59.5%), with 504 patients receiving enteral nutrition (EN) and 214 parenteral nutrition (PN). Early nutrition was more frequently prescribed in the presence of multiple organ failure and was significantly associated with the 28-day mortality in the univariate analysis and propensity-weighted multivariate analysis. Compared with no early nutrition, the association with mortality was strongest with early EN: it was strongest in patients under 65 years with lower SOFA scores. Importantly, the early feeding group included numerous patients with “full feeding”: by day 2 already, the median energy delivery value exceeded 20 kcal/kg.

The observation of the first 10 days’ nutritional management (10 days being the median length of ICU stay) may be considered short though when concluding about the 28-day mortality, as critical care patients’ outcome is influenced by multiple factors. Recently, a group of intensivists published a consensus paper about the essential core outcome measures that must be included for clinical effectiveness trials of nutritional and metabolic interventions in critical illness [2]: one of the agreed issues was that the follow-up should be at least 3 months and if possible 6 months. Indeed, smaller studies with short observation time may provide erroneous information about outcomes. This limitation applies to a study testing in 100 patients the tolerance to early full EN and concluding that the energy supply was optimised by this strategy [3]: but no long-term outcome was provided, while we indeed know from the NUTRIREA-2 study that full early enteral feeding results in more gastrointestinal complications [4]. Others have confirmed that gastrointestinal intolerance occurring during EN was associated with increased mortality [5]. Therefore, the apparent worse outcome of early EN compared to early PN patients in the FRANS study, should not be interpreted as PN being the best option: the problem is just that too much energy was delivered too early, to critically ill patients, which are known to be intolerant to enteral feeding. There are clear indications for PN or supplemental PN [6], and most involve a failing gastrointestinal tract.

Despite being conducted in French-speaking countries, the FRANS study’s observations are probably not country-specific, but time specific. Indeed, the EuroPN observational study [7] was conducted later, in 2019–2020. It included a similar number of patients (*n* = 1172) with detailed nutritional information until day 15, and outcomes were collected until day 90. Feeding ramp-up was clearly part of practice showing integration of more recent recommendations [6]—although with a large variability: also in the EuroPN cohort, many patients received as much as 40 kcal/kg during the first 2 days of their stay, i.e. were overfed in the acute phase. The EuroPN study showed that a feeding dose of 10–20 kcal/kg during the first days was associated with the best outcomes (shortest mechanical ventilation and ICU stay), compared to higher and lower intakes.

Overfeeding is deleterious, and particularly during the first days: in the FRANS study the result was a prolonged mechanical ventilation, the longest being with early EN followed by early PN. The explanation of the poor tolerance to early full feeding is not definitively understood. Nevertheless, the endogenous production of 100–300 g glucose per day [8, 9] which is the physiological response to fasting is highest during the first 72 h and may be a major contributor: it aims at maintaining a continuous blood glucose supply to vital organs. This endogenous glucose production (EGP) is the normal physiological response, but it is unrepressed in critical illness for several days (at least 9 days [9]) despite feeding, i.e. for as long as inflammation persists. If the patient receives feeding amounts exceeding the measured energy expenditure, the organism is not able to handle it [10], and makes no difference between substrates delivered for nutritional or non-nutritional purposes (e.g. sedation lipids or, glucose). Intolerance to overfeeding leading to higher mortality is now well established, especially during the inflammatory phase of disease [11], and should be avoided by any means: this requires real-time monitoring of energy delivery.

The authors conclude that early nutrition support in the ICU was significantly associated with increased 28-day mortality, particularly in younger patients with less severe disease. Although correct in their cohort, this is probably due to “too much too early”. It is important to realise that the FRANS study was conducted in 2015 under the rule of the previous guidelines of the nutrition societies that at that time encouraged feeding as early as possible within the first 48 h with high energy goals of 30 kcal/kg [12–14]. But the guidelines of the European Societies for Intensive Care Medicine (ESICM) [15] and Clinical Nutrition and metabolism (ESPEN) [6] have evolved since, insisting on a cautious progressive ramping-up feeding approach during the first week, and particularly during the first 48 h. The goals during this period should be below 70% of the equation-based targets, and even below the indirect calorimetry measured energy expenditure value [6]. Therefore, the conclusion that their “findings are in contrast with current guidelines on the provision of early nutrition support in the ICU” is not correct: on the contrary, the authors support the evolution towards the actual recommendations.

## Data Availability

Not applicable as there are no data. The comments concern a paper that is accessible on PubMed.
